# Case Report: Probable toripalimab-associated ocular myasthenia gravis-like presentation after chemoimmunotherapy

**DOI:** 10.3389/fneur.2026.1897436

**Published:** 2026-08-31

**Authors:** Hanbing He, Yuan Chen, Li Wang

**Affiliations:** Department of Neurology, Affiliated Hospital of North Sichuan Medical College, Nanchong, Sichuan, China

**Keywords:** case report, immune checkpoint inhibitors, immune-related adverse events, neurotoxicity, ocular myasthenia gravis, toripalimab

## Abstract

Toripalimab, an anti-programmed cell death protein 1 (PD-1) monoclonal antibody developed in China, is increasingly used in the treatment of solid tumors. Although immune checkpoint inhibitors improve antitumor immunity, they may also cause immune-related adverse events involving the nervous system. We report a 60-year-old woman with triple-negative right breast cancer who developed bilateral ptosis and mild limitation of horizontal eye movements approximately 2 weeks after receiving nab-paclitaxel, carboplatin, and toripalimab. Serological testing showed positivity for anti-ryanodine receptor antibody (RYR-IgG, 1:100) and anti-titin antibody (Titin-IgG, 1:320), whereas acetylcholine receptor, muscle-specific kinase, and low-density lipoprotein receptor-related protein 4 antibodies were negative. The neostigmine test was negative. Routine nerve conduction studies were largely normal, and repetitive nerve stimulation showed no low-frequency decrement. After symptom onset, CK-MB mass was markedly elevated despite a normal hs-cTnI level, and AST and ALT were also increased. Because simultaneous total CK and myoglobin measurements were unavailable, the tissue source of the CK-MB elevation could not be determined, and the result was considered uninterpretable in isolation. Possible skeletal-muscle involvement, including ocular myositis, therefore remained in the differential diagnosis. Brain MRI showed chronic ischemic changes without an acute explanatory lesion. No bulbar, respiratory, limb, or cardiac symptoms were documented. After differential assessment, a probable toripalimab-associated ocular myasthenia gravis-like presentation was considered. Toripalimab was discontinued, and oral methylprednisolone 20 mg once daily was administered for 6 days from January 17 to January 22, 2026, beginning the day after discharge. Ptosis improved during early follow-up and remained improved at the subsequent visit. This case illustrates that ocular symptoms after PD-1 inhibitor therapy may represent a neuromuscular immune-related adverse event even when conventional myasthenia gravis antibodies and repetitive nerve stimulation are negative. The significance of RYR-IgG and Titin-IgG positivity in this patient remains uncertain because the assay platform and laboratory cut-offs were unavailable. Their detection should not be regarded as confirmation of ocular myasthenia gravis.

## Introduction

Immune checkpoint inhibitors (ICIs) restore antitumor immune responses and are now used across several malignancies. Toripalimab is a humanized monoclonal antibody targeting PD-1 and has been evaluated in combination regimens for breast cancer (1). With broader clinical use, immune-related adverse events (irAEs) involving endocrine, cutaneous, gastrointestinal, pulmonary, cardiac, muscular, and neurological systems are increasingly encountered in routine practice (2–5).

Neurological irAEs are less frequent than endocrine or cutaneous toxicities, but delayed recognition may lead to serious outcomes. ICI-associated myasthenia gravis (irMG) can overlap with myositis and myocarditis, and ocular symptoms such as ptosis or diplopia may be the initial presentation (6, 7). Diagnosis is difficult when symptoms remain ocular and conventional antibodies or electrophysiological tests are negative. We describe a patient with a probable toripalimab-associated ocular myasthenia gravis-like presentation after chemoimmunotherapy.

## Case description

### General information and medical history

A 60-year-old woman was diagnosed with right breast cancer on November 26, 2025. Histopathological examination of a core needle biopsy from the right breast confirmed invasive carcinoma. Immunohistochemistry showed HER2 negativity (0, no membrane staining), estrogen receptor negative, progesterone receptor negative, Ki-67 approximately 60%, cytokeratin 5/6 scattered positive, p63 negative, p120 membranous positive, and E-cadherin positive. The final diagnosis was invasive carcinoma of no special type of the right breast with moderate nuclear grade. Positron emission tomography-computed tomography showed mildly increased bone metabolism in the left shoulder joint and in several ribs, which was considered more consistent with post-traumatic changes or old fractures than definite metastasis. Although the available imaging showed no definite distant metastasis, a complete TNM stage was not documented in the records available for this report.

Her medical history included hypertension for more than 15 years, with a peak systolic blood pressure of approximately 150 mmHg. She had been taking oral levamlodipine besylate with reportedly adequate blood pressure control. She also had a history of surgical treatment for hemorrhoids and cholelithiasis. There was no known relevant family history.

### Treatment course and symptom onset

On December 10, 2025, the patient received the first cycle of chemoimmunotherapy at Tianjin Cancer Hospital Airport Hospital. The regimen consisted of nab-paclitaxel 300 mg on day 1, carboplatin 150 mg on day 1, and toripalimab 240 mg on day 1. The treating oncologist’s rationale for adding carboplatin was not documented. Platinum-containing regimens have been investigated in triple-negative breast cancer, including in neoadjuvant settings; however, this general evidence does not establish why carboplatin was selected for this patient. The treatment intent and complete TNM stage were also not documented. Approximately 2 weeks after the initial cycle, she developed bilateral ptosis without an identifiable precipitating factor. The ptosis did not show obvious diurnal fluctuation, and she denied diplopia, dyspnea, dysphagia, limb weakness, chest tightness, or palpitations. Because the ptosis persisted, she was admitted on January 15, 2026 for further evaluation and was discharged on January 16, 2026.

### Physical examination

**Figure 1:**
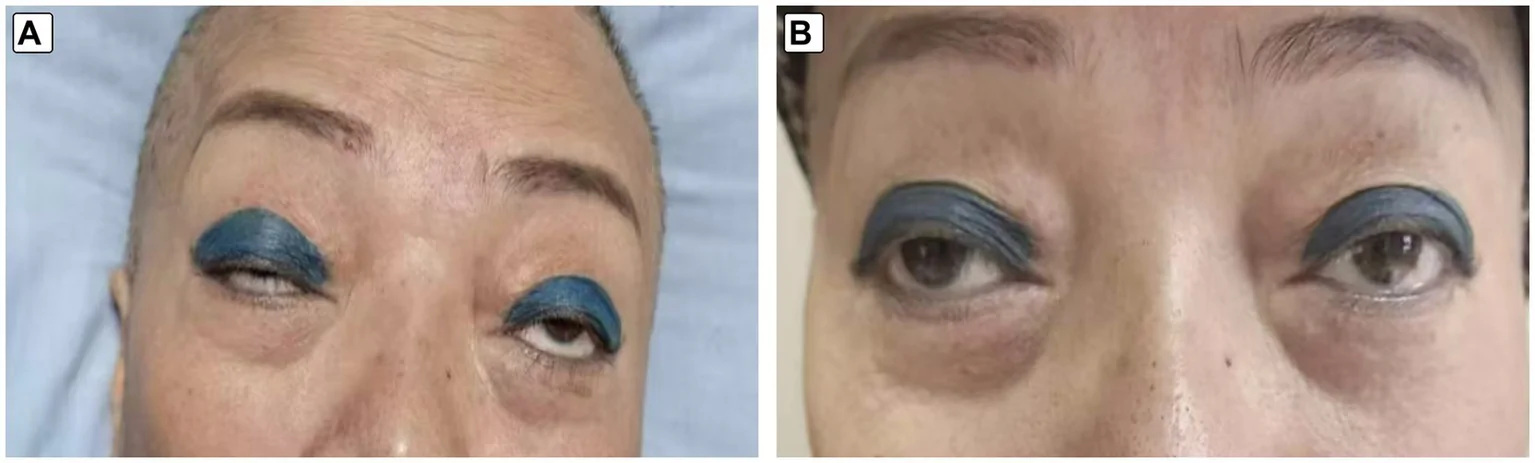
Clinical course of bilateral ptosis before and after treatment. **(A)** Photograph obtained before corticosteroid treatment, showing marked bilateral ptosis. **(B)** Photograph obtained after toripalimab discontinuation and a 6-day course of oral methylprednisolone 20 mg once daily (January 17–22, 2026), showing marked improvement in eyelid opening. Because the photographs were not acquired under standardized conditions, they are intended for qualitative comparison only. The photographs were cropped for presentation; the periocular clinical features were not modified.

On admission, vital signs were stable: body temperature 36.6 °C, pulse 90 beats/min, respiratory rate 20 breaths/min, and blood pressure 126/95 mmHg. Height was 154 cm, weight 55 kg, body surface area 1.53 m^2^, and Karnofsky Performance Status score 100. She was alert and oriented, with clear speech and normal spontaneous respiration. Neurological examination revealed mild limitation of horizontal eye movements and marked bilateral ptosis (Figure 1A). The pupils were equal with brisk light reflexes. Pharyngeal reflex and mastication were normal. Muscle strength was grade 5 in all limbs, muscle tone was normal, and sensation was intact. Deep tendon reflexes were preserved in the upper limbs and absent at the ankles. No pathological reflexes or meningeal irritation signs were present.

### Diagnostic assessment

Serological testing for myasthenia gravis-related antibodies revealed positivity for RYR-IgG (1:100) and Titin-IgG (1:320). AChR-IgG, MuSK-IgG, and LRP4-IgG were negative. The neostigmine test was negative. Motor nerve conduction studies showed normal distal latency, amplitude, and conduction velocity. Sensory nerve conduction was also normal. The tibial nerve H-reflex showed normal latency with mildly decreased amplitude. Repetitive nerve stimulation showed no decremental response during low-frequency stimulation of the right facial, accessory, or ulnar nerves and no incremental response during high-frequency stimulation of the ulnar nerve. The absent ankle reflexes and mildly reduced H-reflex amplitude were considered nonspecific findings of the distal reflex arc. The normal H-reflex latency, otherwise normal motor and sensory nerve conduction studies, and the absence of limb weakness or sensory symptoms did not support a clinically significant peripheral neuropathy. These lower-limb reflex findings could not account for the bilateral ptosis or mild ophthalmoparesis and were therefore considered unrelated to the primary ocular presentation.

Thyroid function tests performed before immunotherapy were within normal limits. Before immunotherapy, non-contrast head CT had shown a possible right basal-ganglia lacunar lesion and nonspecific bilateral periventricular and centrum semiovale white-matter hypodensity, without mass effect or midline shift. These abnormalities therefore predated the ocular symptoms. Chest CT showed the known breast lesion, small inflammatory-appearing pulmonary nodules, scattered fibrotic foci, mild cardiomegaly, and calcified plaques in the aortic wall and left coronary artery. Blood biochemistry on January 15, 2026 showed elevated aspartate aminotransferase (AST, 91 U/L) and alanine aminotransferase (ALT, 144 U/L), without major abnormalities in the complete blood count. Total bile acid was 11.4 μmol/L, and albumin was 39.7 g/L. Abdominal ultrasonography showed hepatic steatosis without intrahepatic or extrahepatic biliary dilatation. Electrocardiography on January 15, 2026 showed sinus rhythm with mild ST-segment depression. Subsequent brain MRI did not reveal an acute lesion that accounted for the bilateral ocular presentation.

The differential diagnosis included an ocular neuromuscular immune-related adverse event, chemotherapy-related neurotoxicity, thyroid-associated ophthalmopathy, cranial neuropathy, and an acute central nervous system lesion. The normal thyroid profile, pupil-sparing bilateral ocular involvement, absence of a single cranial nerve pattern, and brain MRI without an explanatory acute lesion were compatible with an ocular neuromuscular immune-related adverse event. Ocular myositis remained an important competing diagnosis. CK-MB mass was markedly elevated at 93.89 ng/mL (reference ≤5 ng/mL), whereas hs-cTnI remained within the reference range. Because simultaneous total CK, myoglobin, orbital MRI, and other targeted assessments were unavailable, the tissue source of the CK-MB elevation could not be determined, and CK-MB was considered uninterpretable in isolation. The discordant CK-MB and hs-cTnI results, together with the elevated AST and ALT levels, raised the possibility of skeletal-muscle involvement and supported retaining ocular myositis as a competing diagnosis. However, these findings were insufficient to confirm ocular myositis. Baseline transthoracic echocardiography performed before immunotherapy showed preserved left ventricular systolic function (LVEF, 73%), mild left atrial enlargement, and mild mitral regurgitation, without pericardial effusion. Because this examination preceded toripalimab exposure, it could not assess subsequent myocardial involvement. Hepatic steatosis provided an alternative possible explanation for the aminotransferase elevations; therefore, the aminotransferase abnormalities were not specific for skeletal-muscle injury.

### Therapeutic intervention and follow-up

A probable toripalimab-associated ocular myasthenia gravis-like presentation was considered, and further exposure to toripalimab was avoided. Because symptoms were limited to ocular involvement without respiratory, bulbar, or limb weakness, outpatient corticosteroid therapy was recommended with close monitoring. The patient received oral methylprednisolone 20 mg once daily for 6 days, from January 17 to January 22, 2026, beginning the day after discharge. Clinical photographs documented qualitative improvement in eyelid opening during follow-up (Figure 1B). On January 23, 2026, the patient received a modified chemotherapy regimen that excluded toripalimab. At follow-up on February 5, 2026, ptosis remained improved. The improvement could not be attributed solely to methylprednisolone because toripalimab had also been discontinued, time had elapsed, and ocular symptoms may fluctuate spontaneously. The treatment response was therefore considered compatible with, but not confirmatory of, the proposed diagnosis. The clinical course is summarized in Table 1.

**Table 1:** Clinical timeline.

Date/time	Clinical event
November 25, 2025	Baseline thyroid function was within normal limits. Non-contrast head CT showed a possible right basal-ganglia lacunar lesion and nonspecific bilateral periventricular and centrum semiovale white-matter hypodensity, without mass effect or midline shift. Chest CT showed the known breast lesion, small inflammatory-appearing pulmonary nodules, scattered fibrotic foci, mild cardiomegaly, and vascular calcification.
November 26, 2025	Right breast invasive carcinoma was diagnosed by core needle biopsy. Baseline transthoracic echocardiography showed preserved left ventricular systolic function (LVEF, 73%), mild left atrial enlargement, and mild mitral regurgitation, without pericardial effusion.
December 10, 2025	First cycle of nab-paclitaxel, carboplatin, and toripalimab was administered.
Late December 2025	Bilateral ptosis developed approximately 2 weeks after treatment.
January 15, 2026	The patient was admitted for persistent ptosis and ocular movement limitation. AST was 91 U/L and ALT was 144 U/L; electrocardiography showed sinus rhythm with mild ST-segment depression.
January 15–16, 2026	Brain MRI did not reveal an acute lesion that accounted for the bilateral ocular presentation. The patient was discharged.
January 16, 2026	RYR-IgG and Titin-IgG were positive; AChR-IgG, MuSK-IgG, and LRP4-IgG were negative. Neostigmine testing was negative. Nerve conduction studies were largely normal, and repetitive nerve stimulation showed no low-frequency decrement.
January 17–22, 2026	Toripalimab was withheld. Oral methylprednisolone 20 mg once daily was administered for 6 days, beginning the day after discharge.
January 22, 2026	hs-cTnI was 0.047 ng/mL (reference ≤0.053 ng/mL), whereas CK-MB was 93.89 ng/mL (reference ≤5 ng/mL).
January 23, 2026	A modified chemotherapy regimen excluding toripalimab was administered.
After treatment	Clinical photographs documented marked qualitative improvement in eyelid opening.
February 5, 2026	Follow-up showed sustained improvement in ptosis.
February 12, 2026	Repeat cardiac biomarkers showed hs-cTnI 0.004 ng/mL and CK-MB 13.43 ng/mL, decreased from the previous value.

## Discussion

TORCHLIGHT evaluated toripalimab plus nab-paclitaxel in metastatic or recurrent triple-negative breast cancer and did not include carboplatin (1). Platinum-containing regimens have also been investigated in neoadjuvant triple-negative breast cancer (8). This general evidence does not establish the treatment intent or explain why carboplatin was selected for this patient; the regimen should therefore be described as an individualized institutional regimen.

The clinical course was compatible with a probable ICI-associated ocular neuromuscular adverse event rather than typical chemotherapy-related peripheral neurotoxicity. Nab-paclitaxel commonly causes dose-dependent sensory neuropathy after greater cumulative exposure, with paresthesia, numbness, or neuropathic pain rather than isolated bilateral ptosis (9). Platinum agents can also cause predominantly sensory peripheral neurotoxicity, although clinically significant neurotoxicity is less common with carboplatin than with cisplatin (10). In this patient, the ocular symptoms appeared approximately 2 weeks after the first toripalimab-containing cycle and improved after toripalimab withdrawal and corticosteroid treatment. The phenotype and time course were compatible with, but did not confirm, an ICI-associated ocular neuromuscular adverse event.

Diagnostic certainty remained limited. AChR-IgG, MuSK-IgG, and LRP4-IgG were reported as negative, and neither the neostigmine test nor repetitive nerve stimulation provided confirmatory evidence. Because the assay method and laboratory cut-off for AChR-IgG were unavailable, the negative result had limited exclusionary value. These results do not exclude ocular myasthenia gravis, particularly when weakness is confined to ocular muscles. The assessment therefore relied on the ocular phenotype, the temporal association with PD-1 blockade, and the absence of thyroid disease, a single cranial nerve pattern, or an explanatory acute central nervous system lesion. The temporal association supports a probable rather than definite relationship, although the effects of concomitant chemotherapy and spontaneous fluctuation cannot be excluded. Corticosteroids may be considered when ocular symptoms persist or affect daily activities (11, 12).

RYR-IgG and Titin-IgG positivity was noted, but these antibodies do not confirm ocular myasthenia gravis. Striational antibodies have been reported in late-onset and thymoma-associated myasthenia gravis and in some patients with more severe disease (13), but their significance in immune checkpoint inhibitor-associated ocular presentations remains uncertain. In this case, interpretation was further limited by the absence of information on the assay platform, manufacturer, analytical method, and laboratory cut-offs. In addition, RYR-IgG and Titin-IgG were recorded as endpoint titres, an atypical reporting format that further limited their interpretation. Assessment for myositis and myocarditis was warranted because these conditions may overlap with ICI-associated myasthenia gravis, not because the striational antibody results established such overlap.

ICI-associated myasthenia gravis may overlap with myositis and myocarditis (6, 7, 14, 15). The patient had no dyspnea, dysphagia, limb weakness, chest pain, or palpitations, and examination showed no generalized weakness. AST and ALT were elevated, and CK-MB mass was markedly increased to 93.89 ng/mL despite a normal hs-cTnI level. Without simultaneous total CK and myoglobin measurements, CK-MB could not be assigned to either a skeletal-muscle or myocardial source and was uninterpretable in isolation. Nevertheless, the marked CK-MB elevation with normal hs-cTnI, together with the elevated aminotransferase levels, raised the possibility of skeletal-muscle release and strengthened the ocular-myositis differential. These findings did not confirm ocular myositis, and myocardial involvement was not established. Electrocardiography showed mild ST-segment depression, but the available echocardiogram was obtained before toripalimab exposure and could not assess post-onset cardiac involvement. Subclinical skeletal-muscle or myocardial involvement could not be assessed adequately.

The presentation was confined to the ocular muscles, while conventional MG antibodies, neostigmine testing, and repetitive nerve stimulation were negative. Published ICI-associated MG cases often include generalized weakness, myositis, myocarditis, respiratory failure, or anti-AChR antibody positivity (5–7). Isolated ptosis after toripalimab exposure should nevertheless prompt neurological evaluation and assessment for overlapping muscle or cardiac toxicity. Any ICI rechallenge would require an individualized multidisciplinary assessment (16).

This report has several limitations. Single-fiber electromyography was not performed, although it is more sensitive than conventional repetitive nerve stimulation in ocular myasthenia gravis. Sustained upgaze, Cogan lid twitch, ice-pack testing, and a rest test were not systematically recorded. Standardized eyelid measurements were unavailable, and the clinical photographs permit qualitative comparison only. The records did not specify the assay platforms, manufacturers, analytical methods, or laboratory cut-offs for the myasthenia gravis-related antibody tests. In particular, the negative AChR-IgG result had limited exclusionary value because the assay method and cut-off were unknown. RYR-IgG and Titin-IgG were recorded as endpoint titres, an atypical reporting format that further limited their interpretation.

Evaluation for ocular myositis and myocarditis overlap was incomplete. Total CK, myoglobin, NT-proBNP, orbital MRI, post-onset echocardiography, cardiac MRI, continuous rhythm monitoring, and formal respiratory function testing were unavailable. Consequently, the markedly elevated CK-MB could not be assigned to a skeletal-muscle or myocardial source and was uninterpretable in isolation. When considered together with the normal hs-cTnI and elevated AST and ALT levels, the CK-MB result raised the possibility of skeletal-muscle involvement and ocular myositis but could not confirm either. The chest CT report did not specifically assess the thymus or anterior mediastinum, so thymoma was not excluded. Because part of the oncological treatment occurred at another institution, the complete TNM stage, documented treatment intent, and rationale for the carboplatin-containing regimen were not obtained. Finally, this single-patient observation cannot establish a causal relationship between toripalimab and the ocular symptoms.

## Conclusion

This case describes a probable toripalimab-associated ocular neuromuscular immune-related adverse event with a myasthenia gravis-like presentation. Ocular myasthenia gravis could not be confirmed, and ocular myositis was not fully excluded. RYR-IgG and Titin-IgG positivity should be interpreted cautiously and should not be regarded as diagnostic confirmation.

## Patient perspective

The patient was informed of the suspected immune-related nature of her ocular symptoms and the rationale for discontinuing toripalimab. She reported symptomatic relief during follow-up and provided written consent for publication of this case report.

## Data Availability

The datasets presented in this article are not readily available because of ethical and privacy restrictions. Requests to access the datasets should be directed to the corresponding author.
